# Evaluation of birch pollen sensitization profile in allergic rhinoconjunctivitis patients from Bucharest region using component-resolved diagnosis

**DOI:** 10.1186/2045-7022-3-S2-P3

**Published:** 2013-07-16

**Authors:** Florian-Dan Popescu, Mariana Vieru, Adriana Mihaela Tudose

**Affiliations:** 1 University of Medicine and Pharmacy "Carol Davila", Department of Allergology, Bucharest, Romania

## Background

In the Romanian flora, several species of the *Betula* genus are present, especially European white birch (*B. pendula*,syn. *B. verrucosa*) and downy birch (*B. pubescens*,syn. *B. alba*). Rare species are *B. nana* and *B. humilis*. In Southern Romania, the sensitization to *Betulaceae* pollen in patients with allergic rhinitis is less frequent than that to *Poaceae* pollen. The flowering season of the birch trees starts mostly in early April and lasts until mid of May, while the grass flowering period starts in May and finishes at the mid of July.

## Methods

We enrolled subjects from Bucharest and the surrounding counties in central part of Southern Romania, presenting symptoms of rhinoconjunctivitis in early May (with or without symptoms earlier in April) and having positive skin prick tests to birch or *Betulaceae* tree pollen extracts, with or without positive skin prick tests to mixed grass pollen extract or *Phleum pratense* pollen extract. Serum levels of specific IgE to birch pollen (t3), Timothy grass pollen (g6), and to recombinant allergen components Bet v 1, Bet v 2, Bet v 4, Bet v 6, Phl p 1, Phl p 5, Phl p 7, Phl p 12 were measured using a novel multiparameter immunoblot test system based on single purified allergen components (SPAC Pollen).

## Results

Sera were collected from fourteen patients presenting positive skin prick tests to *Betulaceae* mixed pollen extract (100 IR/mL) and/or *Betula alba* pollen extract (100 IR/mL). We found 85.71% patients with specific IgE against Bet v 1 (genuine sensitization to birch pollen). For the rest, specific IgE to *Phleum* specific allergen components (Phl p 1, Phl p 5) were detected, while the birch-specific marker Bet v 1 being not recognized by IgE antibodies. Moreover, 21.42% of all subjects were co-sensitized to birch and grass pollen based on the presence of specific IgE to Phl p 1, Phl p 5 and Bet v 1.

## Conclusion

Because birch trees and grasses may have a temporal overlap in flowering periods in some regions, and their pollen grains contain cross-reactive allergen components (profilins Bet v 2 and Phl p 12, polcalcins Bet v 4 and Phl p 7), the *in vitro* diagnostic testing with the marker allergens Bet v 1, Phl p 1, Phl p 5 allows the clinician to confirm true sensitization to the corresponding allergen plant sources for an accurate prescription of allergen-specific immunotherapy. This is particularly important for Southern Romania, a region with a great plant biodiversity.

